# Caffeine suppresses homologous recombination through interference with RAD51-mediated joint molecule formation

**DOI:** 10.1093/nar/gkt375

**Published:** 2013-05-10

**Authors:** Alex N. Zelensky, Humberto Sanchez, Dejan Ristic, Iztok Vidic, Sari E. van Rossum-Fikkert, Jeroen Essers, Claire Wyman, Roland Kanaar

**Affiliations:** ^1^Department of Cell Biology and Genetics, Cancer Genomics Center, Erasmus Medical Center, PO Box 2040, 3000 CA, Rotterdam, The Netherlands, ^2^Department of Radiation Oncology, Erasmus Medical Center, PO Box 2040, 3000 CA, Rotterdam, The Netherlands and ^3^Department of Vascular Surgery, Erasmus Medical Center, PO Box 2040, 3000 CA, Rotterdam, The Netherlands

## Abstract

Caffeine is a widely used inhibitor of the protein kinases that play a central role in the DNA damage response. We used chemical inhibitors and genetically deficient mouse embryonic stem cell lines to study the role of DNA damage response in stable integration of the transfected DNA and found that caffeine rapidly, efficiently and reversibly inhibited homologous integration of the transfected DNA as measured by several homologous recombination-mediated gene-targeting assays. Biochemical and structural biology experiments revealed that caffeine interfered with a pivotal step in homologous recombination, homologous joint molecule formation, through increasing interactions of the RAD51 nucleoprotein filament with non-homologous DNA. Our results suggest that recombination pathways dependent on extensive homology search are caffeine-sensitive and stress the importance of considering direct checkpoint-independent mechanisms in the interpretation of the effects of caffeine on DNA repair.

## INTRODUCTION

Gene targeting (GT) by homologous recombination (HR) is a genetic tool of unrivaled power and flexibility (1,2) that was instrumental in the development of the famous double-strand break (DSB) model of HR (3,4). The technique is efficient and straightforward in model yeast species (*Saccharomyces cerevisiae* and *Schizosaccharomyces pombe*) (5), some protists (6), a plant species (7) and also practical in several vertebrate cell lines (8–11), of which mouse embryonic stem (ES) cells are arguably the most important, as they allowed and popularized performing reverse genetics studies in a mammalian model organism.

Why exactly GT is more efficient in some cells than the others is unclear. Part of the difference can be attributed to the relative activities of the DNA repair systems that are responsible for stable integration of the exogenous DNA. Yeast relies primarily on HR for DSB repair, whereas in higher eukarya, homology-independent mechanisms play a more prominent role. However, HR proteins are ubiquitously expressed in animal tissues and are essential for the viability of proliferating cells. On the other hand, there are numerous differences in the biology between GT-proficient and GT-refractory cells, which may include the mechanism of delivery of the exogenous DNA, cellular response to its presence, its persistence and processing within the cell.

DSBs in genomic DNA are critical lesions for dividing cells, and elaborate signaling systems, the DNA damage response (DDR), have evolved to detect them and delay the cell cycle progression until they are repaired (12,13). Protein kinases ATM, ATR and DNA-PK—members of the phosphatidylinositol 3-kinase-related kinase (PIKK) superfamily (14)—play a central role in this process. As progression through mitosis with even a single unrepaired DSB will lead to the loss of genetic information, the DDR system is extremely sensitive. A single DSB (linear microinjected DNA, unprotected telomere) can trigger DDR signaling leading to cell cycle arrest in a eukaryotic cell (15,16). As GT involves introduction of large numbers of linear DNA molecules into the cell, it is natural to expect a similar reaction to the transfected DNA, which in turn may affect whether and how it integrates into the genome.

Surprisingly little is known about the DDR to DNA transfection (17), and to the best of our knowledge, this was not studied in the context of GT at all. Noteworthy is a reverse correlation between the efficiency of GT—both absolute and relative—and the efficiency of transfection; with microinjection, which delivers defined and relatively small number of DNA molecules into the nucleus, the absolute frequency of GT is on the order of 10^−^^3^, whereas it is ∼10^−^^7^ for mass-delivery methods (electroporation, lipofection) (1,18). The aim of our study was to investigate the potential involvement of DDR in GT and random integration of the targeting DNA constructs in mouse ES cells.

## MATERIALS AND METHODS

### Cell lines and culture

IB10 (a subclone of the E14 line, 129/Ola) and other mouse ES cells used in the study were grown on 0.1% gelatin-coated plastic dishes in 1:1 mixture of Dulbecco’s modified Eagle’s medium (DMEM) (Lonza BioWhittaker Cat. BE12-604F/U1, with Ultraglutamine 1, 4.5 g/l Glucose) and buffalo rat liver cell (BRL)-conditioned DMEM, supplemented with 1000 U/ml leukemia inhibitory factor, 10% FCS, 1× NEAA, 200 U/ml penicillin, 200 µg/ml streptomycin and 89 µM β-mercaptoethanol. HT1080 cells were grown in DMEM media (high glucose, 1 mM sodium pyruvate, L-glutamine) supplemented with 10% FCS, 2× NEAA, 100 U/ml penicillin and 100 µg/ml streptomycin. H2AX−/− (H2afx) ES cell line was kindly provided by Andre Nussenzweig (19); DNA-PKcs−/− (Prkdc) and Rad54−/− lines were described previously (20,21). The p53−/− (Trp53) ES cells were isolated *de novo* from the mouse line originally generated by Jacks *et al.* (22). HT1080 cells were grown in HAT medium (0.1 mM hypoxanthine, 0.4 µM aminopterin, 16 µM thymidine in HT1080 growth medium) for two passages and in HT medium for two days before the experiment to eliminate background HPRT-negative cells.

### GT and random integration assays

The Rad54-GFP.puro and Rosa26-βgeo targeting constructs were described previously (23,24). After linearization with PvuI (Rad54) or NotI (Rosa26), the plasmid DNA was extracted with phenol-chloroform, precipitated and dissolved in deionized water. In some experiments, 2 µg of linearized pBS-PGK-puro construct was added to 10 µg of linearized Rosa26-βgeo to monitor random integration frequency based on the frequency formation of puromycin-resistant colonies.

For a typical Rad54-GFP and Rosa26-βgeo GT assay, exponentially growing ES cells were trypsinized, collected by centrifugation and dissolved in ES growth media at 1–1.5 × 10^7^/ml. In all, 480 µl of the suspension was transferred into a 2 mm gap electroporation cuvette (BTX Harvard Apparatus Model No 620), mixed with 10 µg of linearized targeting construct DNA and electroporated using GenePulser Xcell apparatus (118 V, 1200 µF, ∞ Ω, exponential decay). Electroporated cells were seeded at 2–3 × 10^6^ per gelatinized 10 cm dish, and antibiotic selection was started the day after. In the Rad54-GFP GT assay, selection with 1.5 µg/ml puromycin was maintained for 6 days, after which the stably transformed cells were trypsinized, collected by centrifugation, fixed with 1 ml of 1% paraformaldehyde in phosphate buffered saline (PBS) for 15 min and analyzed by fluorescence-activated cell sorting (FACS) after addition of an equal volume of 0.2% Triton X100 in PBS (fixation and detergent improve the separation between Rad54-GFP positive and negative cell populations). Cells targeted with Rosa26-βgeo were selected with 200 µg/ml G418 for 8 days, resistant colonies were fixed, stained and counted. The G418-resistant colony numbers were normalized to viability measured in the same conditions by colony formation assay.

The effect on random integration was independently assessed by electroporating the cells with circular or DraIII-linearized pEGFP-N1 plasmid in the same conditions as used for the GT assays. Several dilutions of the electroporated cells were seeded for plating efficiency estimation, whereas the rest were seeded at 0.5–1 × 10^6^ per 10 cm dish and selected with 200 µg/ml G418.

For transfection HT1080 cells were resuspended in growth medium at 7 × 10^6^/0.5 ml, transferred into 2 mm gap electroporation cuvette and eclectroporated using GenePulser Xcell (BioRad) apparatus at 200 V, 250 µF, ∞ Ω, exponential decay with SalI-linearized pHPRThyg targeting construct (25). Several electroporation reactions were pulled together. Following the electroporation, 200 or 1000 cells were seeded into non-selective media for plating efficiency determination, whereas the rest were divided into several 10 cm dishes to measure random integration frequency by selection with hygromycin B, GT frequency by combined hygromycin B and 6-thioguanine selection. Caffeine treatment was started after plating and maintained overnight. Selection with hygromycin B (100 µg/ml) and 6-thioguanine (30 µg/ml) was started 1 and 5 days after transfection, respectively. Colony counts were adjusted for the effect of caffeine on plating efficiency.

### Inhibitors

Stock solutions used were 40 mM caffeine in ES media (most experiments); 100 mM xanthines (caffeine, theophylline, theobromine, pentoxifilline, hypoxanthine, xanthine) in 0.1 M NaOH; 10 mM forskolin in 95% ethanol; 50 mM roscovitine in dimethyl sulfoxide (DMSO), UCN-01 (Sigma, U6508) 100 µM in DMSO, VE-821 (Axon Medchem) 10 mM in DMSO.

### FACS and antibodies

Two-parameter cell cycle distribution analysis was performed as described (26), but at the counterstaining step, the concentration propidium iodide (PI) was 2 µg/ml, and the concentration of RNase was 0.25 mg/ml. PAGE electrophoresis and transfer to nitrocellulose membrane for western blotting was performed following standard protocols. The membranes were blocked with 5% skim milk in PBS + 0.04% Tween20 and probed with the following antibodies: Chk1 [mouse mAb (G4), Santa Cruz], Chk2 (mouse mAb, BD Bioscience), phospho-Chk1(S345) [rabbit mAb (133D3), Cell Signaling].

### Recombinant proteins, electrophoretic mobility shift assay and D-loop assays

Human RAD51 and RPA were expressed in *Escherichia coli* and purified as described (27,28). The MRE11/RAD50/NBS1 complex was purified from baculo virus infected Sf9 cells as described (29).

Recombinant RAD51 was incubated in a final volume of 10 μl with 5′-end Alexa Fluor 488-labeled 90 nt single-stranded DNA (ssDNA) (30) (AF488SK3ss) at 3.6 μM (nt), in reaction buffer [50 mM Tris–HCl (pH 7.5), 1 mM DTT, 0.1 mg/ml acetylated BSA, 60 mM KCl, 2 mM CaCl2 and 1 mM ATP] (31). After 5 min incubation at 37°C, 2 μl of supercoiled pUC19 plasmid DNA at 0.8 mg/ml (purified by double CsCl density gradient centrifugation) and 2 μl of the indicated drug (caffeine or xanthine, final concentrations: 0, 1.25, 2.5, 5 and 10 mM, respectively) were added and further incubated for 20 min at 37°C. Samples were deproteinized by adding SDS (1%), EDTA (25 mM) and Proteinase K (1 μg/μl) and incubated for 5 min at 37°C. Reaction mixtures were resolved by 0.7% agarose gel electrophoresis in 0.5×TB buffer. Gels were analyzed using a Typhoon Trio scanner exciting the dye-coupled DNA with a 488 nm laser and detecting emission intensity with a 520 nm BP40 filter at 600 V PMT, 3 mm focal plane. Images obtained were analyzed with ImageQuant version 5.2 (Molecular Dynamics).

Reaction mixtures for electrophoretic mobility shift assays (EMSA) with RAD51 containing AF488SK3ss (90 nM in nt) or AF488SK3ds (90 nM in bp) and human RAD51 (300 nM) were assembled in reaction buffer in the presence of caffeine (final concentrations: 0, 0.5, 1, 5 and 10 mM, respectively). Samples were incubated for 10 min at 37°C, in a final volume of 20 μl. The reactions products were separated on a 5% non-denaturing PAGE running in 0.5× TB buffer at 4°C. The labeled DNA was visualized by direct scanning as described earlier in the text.

Binding reactions with RPA and MRN for EMSA were performed in a volume of 20 μl for 15 min at 20°C and contained the indicated concentrations of proteins, 1 nM 70 nt ssDNA labeled with Alexa Fluor 532 at 5′site, 30 mM Tris–HCl (pH 7.5), 30 mM NaCl, 1 mM DTT and 5% glycerol. Reaction mixtures were electrophoresed through native 5% PAGE (30:1; 0.5× TBE) at 4°C for 4 h at 20 V/cm.

For intercalation tests, circular plasmid DNA (pGATC, 3199 bp) was singly nicked by incubation with DNase I for 30 min at 30°C in the presence of 0.36 mg/ml ethidium bromide [5× nicking buffer: 100 mM Tris–HCl (pH 7.5), 250 mM NaCl, 1.8 mg/ml EtBr, 50 mM MgCl2; reaction: 0.16 µg/ml DNA, 1× nicking buffer, 50 µg/ml BSA (32)]. Optimal concentration of DNase I was determined by titration and was 3 µg/ml. The reaction was stopped by addition of 1:10 volume stop mix (5% SDS, 50 mM EDTA). Single-nicked plasmid was phenol:chloroform extracted, precipitated and dissolved in water. In all, 18.75 ng/µl of nicked plasmid was incubated in the presence of different concentrations of intercalating agents in 1× T4 ligase buffer (Roche) for 30 min at room temperature, after which the ligase was added, and ligation was performed at 16°C for 1 h. For topoisomer separation, the ligated plasmids were phenol:chloroform extracted and precipitated, dissolved in water and separated on 1.3% agarose gel prepared with running buffer (1× TBE, 0.3 µg/ml chloroquine) for 44 h at 4°C, 2.5 V/cm.

### Scanning force microscopy

Three′-tailed DNA was made as previously described (33,34) using plasmid DR6 (34) as a template DNA for PCR. Linear DNA with blunt ends was produced by cutting plasmid DR6 with SmaI and EcoRV. The products of double digestion were resolved on 1% agarose gel. In all, 693 bp blunt-ended DNA fragment was purified from gel using GFX column. Plasmid DR6 was used as supercoiled circular homologous DNA. Plasmid pDERI1 (33) was used as supercoiled circular heterologous DNA.

Human RAD51-DNA complexes were formed in 10 µl of reactions containing 7.5 μM DNA (concentration in nt/bp, adjusted concentration to indicate nt or bp along the DNA such that RAD51 concentration is sufficient to cover the entire DNA fragment at one monomer per 3 nt or bp), 2.5 μM RAD51, 25 mM HEPES-KOH (pH7.5), 5 mM CaCl_2_, 2 mM ATP, 30 mM KCl ± 4 mM caffeine. Reactions were incubated at 37°C for 30 min and than placed on ice. Reactions were diluted 15-fold in deposition buffer [10 mM HEPES-KOH (pH 7.5), 10 mM MgCl_2_] and deposited on freshly cleaved mica. After 15 s, the mica was washed with water and dried in a stream of filtered air. Joint molecules were formed and deposited for imaging as follows: One-fifth of the volume of the filament formation reaction mixture was mixed with 0.75 μM homologous or heterologous circular DNA (concentration in bp), in 25 mM HEPES-KOH (pH7.5), 5 mM CaCl_2_, 2 mM ATP, 30 mM KCl ± 4 mM caffeine. After 30 min at 37°C, 100 mM (NH4)_2_SO_4_ (final concentration) was added and incubated for 10 min at 37°C. This step disrupts possible non-specific aggregation of RAD51 filaments and DNA. The reaction mixture was transferred onto freshly cleaved mica and after 20 s washed with 10 mM HEPES-KOH (pH 7.5), 50 mM KCl. The excess buffer was removed and replaced with a buffer containing 10 mM HEPES-KOH (pH7.5), 10 mM MgCl_2_ for DNA to attach to mica. After 5 s, the mica was washed with water followed by drying with a stream of filtered air.

Images were obtained with a NanoScope IV SFM (Digital Instruments; Santa Barbara, CA) operating in tapping mode in air with a type E scanner. Uncoated silicon Pointprobe tips were type NHC-W, resonance frequency 310–372 kHz, force constant C = 29.0–52.0 N/m, (Nanosensors supplied by Veeco Instruments, Europe). The length measurements were done from NanoScope images imported into IMAGE SXM 1.62 (NIH-IMAGE version modified by Steve Barrett, Surface Science Research Centre, Univ. of Liverpool, Liverpool, UK). The contours of filaments and DNA were traced manually.

## RESULTS

### Caffeine efficiently inhibits GT

**Figure 1.**
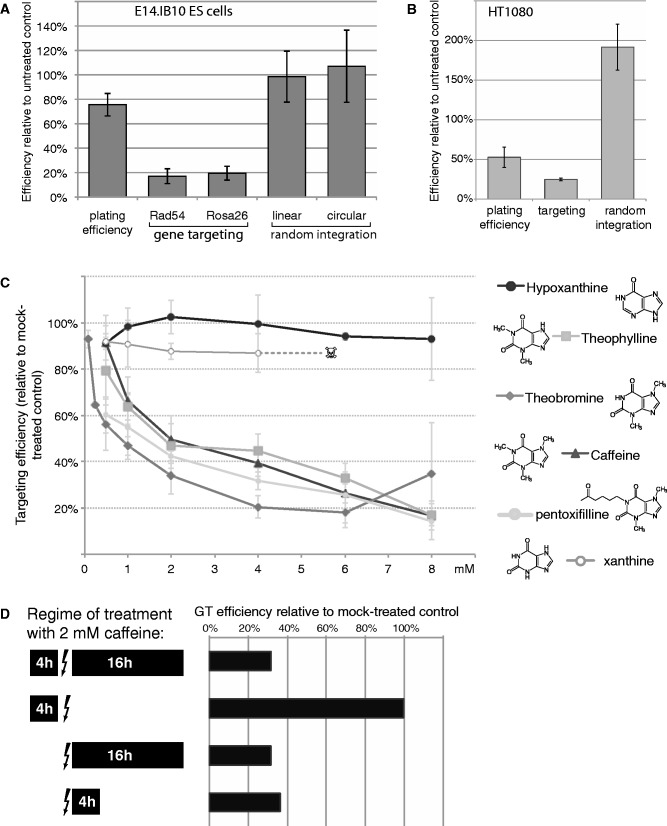
Inhibitory effect of caffeine on GT. (**A**) Caffeine inhibits GT in ES cells with little effect on random integration of a linear or circular plasmid. IB10 ES cells were electroporated with a targeting construct (Rad54-GFP or Rosa26-ßgeo) or linear or circular pEGFP-N1 plasmid, plated into growth media containing 4 mM of caffeine and let recover overnight. Caffeine-containing media was replaced with fresh media after 16 h, and selection was started 20–24 h after electroporation and maintained for 6–8 days. Viability, indicated as plating efficiency relative to untreated control, was determined in a colony formation assay under the same conditions. The mean of three independent experiments is plotted, error bars indicate 1 SD. (**B**) Effect of caffeine on GT in the human fibrosarcoma cell line HT1080. (**C**) Effect of overnight (∼16 h) treatment with different concentrations of caffeine and related compounds on GT efficiency measured by the Rad54-GFP assay. The mean of three independent experiments are plotted with the error bars indicating 1 SD. (**D**) Effect of 2 mM caffeine present for different periods before and after electroporation (indicated by arrow) of the Rad54-GFP targeting construct on GT efficiency.

To measure the efficiency of GT, we used the Rad54-GFP (23) and Rosa26-ßgeo (24) GT assays in ES cells. In both assays, stable transformants produced by HR at the target locus are detected based on the expression of a reporter protein (Rad54-GFP or β-galactosidase-*neo*, respectively) from the promoter in the targeted locus (knock-in) (Supplementary Figure S1). To test the possible involvement of the DDR in stable cell transformation, we treated mouse ES cells with caffeine aiming to inhibit the ATM and ATR kinases. When added after electroporation with the targeting construct and kept overnight (∼16 h) 4 mM of caffeine strongly (∼80%) inhibited GT at the Rad54 and Rosa26 loci while having no negative effect on random integration of a linear or circular plasmid DNA and only mildly impairing clonogenic survival (Figure 1A). The effect was not limited to mouse cells, as ∼75% inhibition of the HPRT locus targeting (18) was observed in the human fibrosarcoma cell line HT1080 (Figure 1B).

We next determined whether the effect on GT was caffeine specific by testing several related compounds; theophylline, theobromine, pentoxifylline, xanthine and hypoxanthine. Theophilline (1,3-dimethylxanthine) and theobromine (3,7-dimethylxanthine), which lack one methyl group compared with caffeine (1,3,7-trimethylxanthine), have similar effects on checkpoint activation (35,36) and in our assays inhibited GT similar to caffeine (Figure 1C). In contrast, hypoxanthine and xanthine had no negative effect on GT (Figure 1C). GT inhibition by theobromine was somewhat more efficient than by the other two alkylxanthines, an effect equivalent to 1 mM caffeine was observed with 0.25 mM theobromine.

Treatment with methylxanthines never resulted in GT efficiency inhibition below 17–20%; therefore, a fraction of GT events may be insensitive to the treatment. Pre-incubating or including caffeine into the electroporation mixture to ensure continuous exposure did not eliminate the caffeine-resistant fraction of the GT events (Figure 1D and data not shown). Comparison of the effect of different caffeine treatment regimes on GT also suggests that caffeine acts as a direct chemical inhibitor of the GT process, and that GT, or at least its caffeine-sensitive step(s), is complete within ∼4 h after the electroporation. If caffeine acted indirectly, for example by altering HR protein concentrations, cell cycle progression or genomic DNA integrity, pre-treatment with caffeine would be expected to produce some negative effect.

### Inhibition of GT by caffeine is not mediated by affecting checkpoints

**Figure 2.**
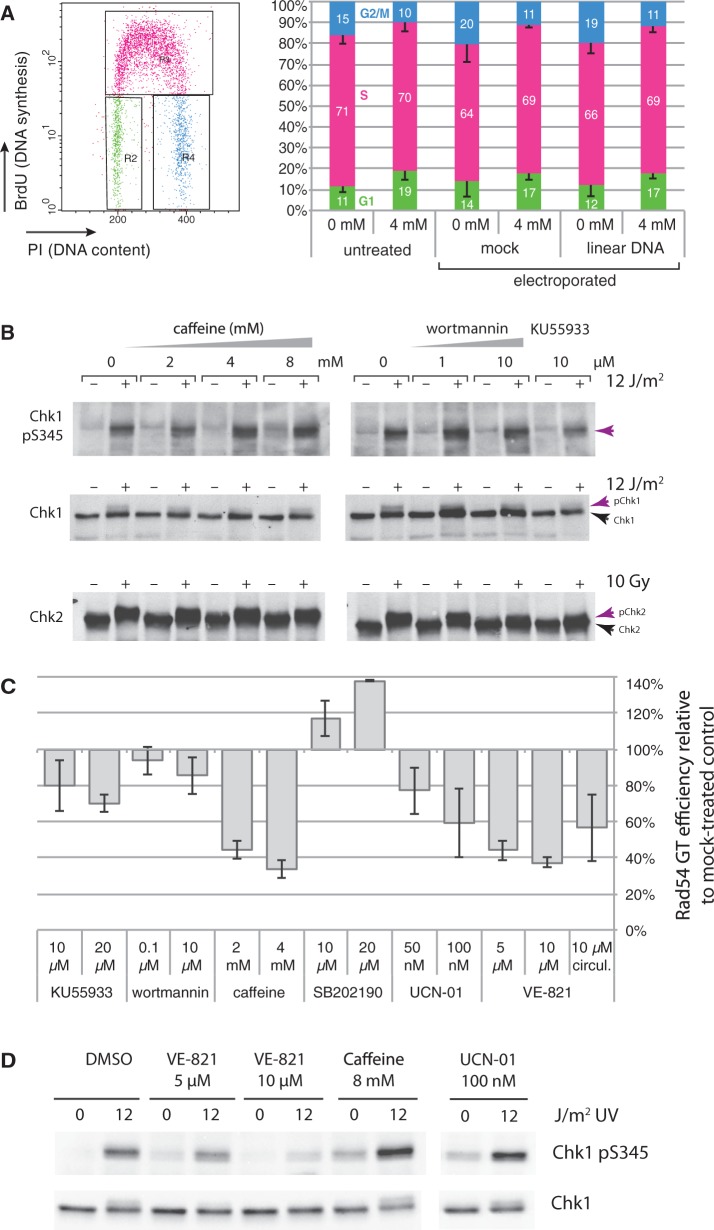
GT inhibition by caffeine is not mediated by the effect on checkpoint kinases. (**A**) Effect of caffeine on cell cycle distribution. IB10 cells were treated for 4 h with 0 or 4 mM caffeine in three conditions; exponentially growing on plastic, mock electroporation and electroporation with 10 µg Rad54-GFP targeting construct (i.e. as in a typical targeting experiment). BrdU was added to the media in the final 10 min of the incubation to label newly synthesized DNA. Cells were fixed, stained with PI and anti-BrdU-Alexa488 antibody and analyzed by FACS; gates used for cell-cycle phase distribution assignment are shown on a representative plot at the left. (**B**) Caffeine treatment does not suppress the phosphorylation of the downstream ATM and ATR targets. Exponentially growing IB10 cells were treated with the indicated concentrations of the inhibitors for 3 h, irradiated (10 Gy γ-ray or 12 J/m^2^ UV or mock) and incubated for another 4 h in the presence of the inhibitor. For UV irradiation, the media was removed and reserved, and cells were washed with PBS before irradiation; the media was returned after irradiation. Cells were then lysed in Laemmli sample buffer and analyzed by western blotting with the indicated antibodies. (**C**) Effect of specific inhibitors of PIKK on GT at Rad54 locus. IB10 cells were incubated in the presence of the inhibitor for 4 h after the electroporation of the Rad54-GFP-targeting construct. The targeting efficiency normalized to vehicle-treated control is shown. (**D**) Effect of ATR and Chk1 inhibitors (VE-821 and UCN-01, respectively) on UV-induced Chk1 phosphorylation. Cells were washed with PBS, exposed to 12 J/m^2^ UVC, covered with media containing the indicated concentration of the inhibitor, incubated for 1 h and lysed.

We took several approaches to determine whether GT suppression by caffeine results from the inhibition of PIKK kinases. We used other—more specific—chemical inhibitors (KU55933, wortmannin, VE-821 and UCN-01), ES cell lines deficient for the key proteins involved in the DDR (DNA-PK, p53, H2AX) and monitored changes in cell cycle and checkpoint signaling activity in response to caffeine treatment. Treatment with caffeine alone had little effect on the cell cycle distribution as determined by two parameter FACS analyses (Figure 2A). In exponentially growing ES cells, treatment with 4 mM caffeine for 4 h led to an increase in the size of the G1 cell fraction (11–19%) at the expense of the G2/M-phase fraction, regardless of whether the targeting construct DNA was present. As even in the presence of caffeine, ∼80% of the population remains in the HR-permitting S and G2/M phases, the observed cell cycle redistribution cannot explain the GT suppression.

Caffeine in a concentration range of 1–4 mM is used to inhibit the PIKK checkpoint kinases (ATR, DNA-PK and ATM), including in studies using mouse ES cells (37). However, it is unclear whether PIKK inhibition is the sole culprit of radiosensitization and whether it efficiently occurs *in vivo* in cells (37–40). To test the status of the ATM and ATR kinases under our conditions, we investigated the phosphorylation of their downstream targets Chk2 and Chk1 in response to ionizing or UV irradiation, respectively (Figure 2B and D). Cells were pre-treated with the inhibitors for 3 h, irradiated, grown in the same media for 1 h more and lysed. SDS–PAGE mobility of the ATM target Chk2 was reduced in response to 10 Gy ionizing radiation, indicative of the Chk2 phosphorylation. The reduction in mobility was suppressed by the treatment with the specific ATM inhibitor KU55933, and wortmannin at 10 µM. In contrast, the 2–4 mM of caffeine had little or no effect on Chk2 phosphorylation. UV-induced phosphorylation of Chk1, which in the DDR is completely dependent on ATR (39) and was fully suppressed by the ATR inhibitor VE-821(Figure 2D), was not suppressed by caffeine. On the contrary, phosphorylation of serine 345 increased in caffeine-treated UV-irradiated ES cells in a dose-dependent manner. This is in line with the previous observations made with human somatic cells (39), where hyperphosphorylation of Chk1 and other ATR and ATM substrates occurs in response to hydroxyurea (HU) treatment in the presence of 1–8 mM caffeine and with the caffeine dose-dependent increase in endogenous γH2AX in mouse ES cells (41). As we found that the concentrations of caffeine that suppress most of the GT events in the same treatment regime have no negative effect on ATM and ATR activity, caffeine must be affecting GT via a different mechanism. Theobromine, which inhibited GT at lower concentrations than caffeine, did not inhibit UV-induced Chk1 phosphorylation either (data not shown).

We then studied the effect of KU55933 and wortmannin on GT. As 4 h incubation with caffeine after electroporation had almost the same inhibitory effect on GT as overnight incubation (Figure 1D), we used this shorter exposure regime. KU55933 slightly decreased the GT efficiency when present during the first 4 h after electroporation (Figure 2C). Importantly, the same concentration of the compound nearly completely blocked the phosphorylation of ATM’s downstream target Chk2 (Figure 2B). Wortmannin, which has highest specificity for DNA-PK [*in vitro* IC50 DNA-PK 16 nM, ATM 160 nM, ATR 1.8 µM (42)], also reduced GT efficiency by up to 15%. As the Rad54-GFP-targeting assay measures relative GT efficiency, which is negatively affected by both decreases in GT and by DNA-damage induced increases in random integration, the observed small negative effect may be due to increased genomic instability in the presence of the inhibitors. In addition to Chk1 and Chk2, recently a third effector pathway has been defined downstream of ATM and ATR, mediated by p38MAPK kinase (MAPK14) (43), but its specific inhibitor SB202190 had a positive rather than negative effect on GT (Figure 2C).

Although caffeine did not inhibit ATR-mediated phosphorylation of Chk1 in mouse ES cells, we nevertheless used the inhibitors of ATR-Chk1 pathway to test whether it has an impact on GT. ATR inhibitor VE-821 (44) at 5–10 µM strongly suppressed GT, and Chk1 inhibitor UCN-01 (45) had a similar effect but to a lesser extent. There was a correlation between the efficiency of inhibition by VE-821 of GT and of UV-induced phosphorylation of Chk1, indicating that the effect is ATR-specific (Figure 2C and D). To test whether the ATR signal, that GT depends on, is triggered by the transfected DNA, we substituted linear targeting construct for its circular version. The latter is much more stable in the cell, is expected to induce lower DDR activation and thus be immune to DDR inhibition. GT with circular DNA was still suppressed by VE-821.

If the DDR is involved in stable transformation and mediates the inhibitory effect of caffeine on GT, cells deficient in the major DDR components are expected to have altered GT efficiencies and be less responsive to caffeine. To test this possibility, we measured the efficiency of GT using Rad54-GFP assay in ES cell lines deficient for p53, H2AX or DNA-PKcs. We also analyzed the effect of caffeine in a cell line deficient for Rad54 and thus characterized by reduced HR efficiency. In all the cell lines tested, caffeine treatment reduced the relative GT efficiency by ∼80% (Table 1)*.* Inhibition of GT by caffeine in p53−/− cells is particularly important, as several signaling pathways affected by caffeine are mediated by p53 (46).

**Table 1.** Inhibition of GT by caffeine in knock-out ES cell lines measured by Rad54-GFP assay

Cell line	GT efficiency at 4 mM caffeine, normalized to 0 mM (standard deviation)
p53−/−	20% (4%)
Rad54−/−	18% (3%)
H2AX−/−	26% (5%)
DNA-PK−/−	20% (7%)

Numbers represent three independent experiments. Treatment with 4 mM caffeine was started after the electroporation of the targeting construct and maintained for ∼16 h. GT efficiency in the plates treated with 4 mM caffeine was normalized to untreated control plates from the same experiment.

Taken together, the experiments with specific ATM and DNA-PK inhibitors and knock-out ES cell lines, and the lack of cell cycle perturbations in transfected cells, suggest that major components of the DDR are dispensable for GT and that response to the transfected DNA plays a small role in the stable transformation pathway selection, if any at all.

### GT inhibition is not due to other known effects of caffeine

**Figure 3.**
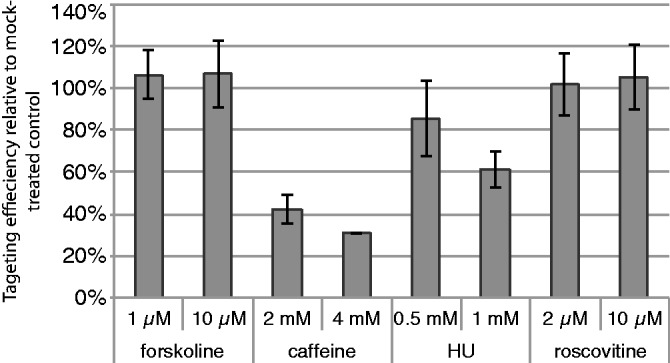
Other effects of caffeine. Emulating other possible effects of caffeine (increase in cAMP, suppression of DNA synthesis, inhibition of CDK) with alternative treatments (forskolin, HU, roscovitine). IB10 cells were electroporated with the Rad54-GFP targeting construct and plated in the medium containing indicated concentrations of the drugs. After 4 h, the medium was replaced with the drug-free (cells that were not yet attached were collected by centrifugation and returned to the plate).

A 4-h treatment with 2 mM caffeine causes the inhibition of CDK2 activity in ES cells (37). HR is a cell-cycle-regulated process, and several mechanisms of this regulation by cyclin-dependent kinases have recently emerged; CDK phosphorylation of CtIP is required for DSB end resection (47), CDK phosphorylation of BRCA2 C-terminal domain regulates its interaction with RAD51 (48). To test whether CDK2 or other CDK inhibition mediates GT suppression, we treated IB10 cells with 2 or 10 µM roscovitine, a reversible selective cell permeable inhibitor of CDK1 (IC50 0.65 µM), CDK2 (IC50 0.7 µM) and CDK5 (IC50 0.16 µM) (49), for various periods of time before and after the electroporation of the Rad54-GFP targeting construct. Roscovitine treatment within 4 h after electroporation, a period where inhibition by caffeine is most efficient, did not have a negative effect on GT (Figure 3). Inhibition of DNA synthesis (repair or replication) is another previously demonstrated effect of caffeine (50), which could contribute to its suppression of GT. However, in our experiments, caffeine had a small effect on BrdU incorporation (Figure 2A), whereas treating cells with 0.5 or 1 mM HU, which causes a near-complete replication block (85 and 97% reduction in BrdU+ cells, respectively), had a much lower effect on GT (Figure 3 and data not shown).

### Evidence for direct inhibition of HR by caffeine

**Figure 4.**
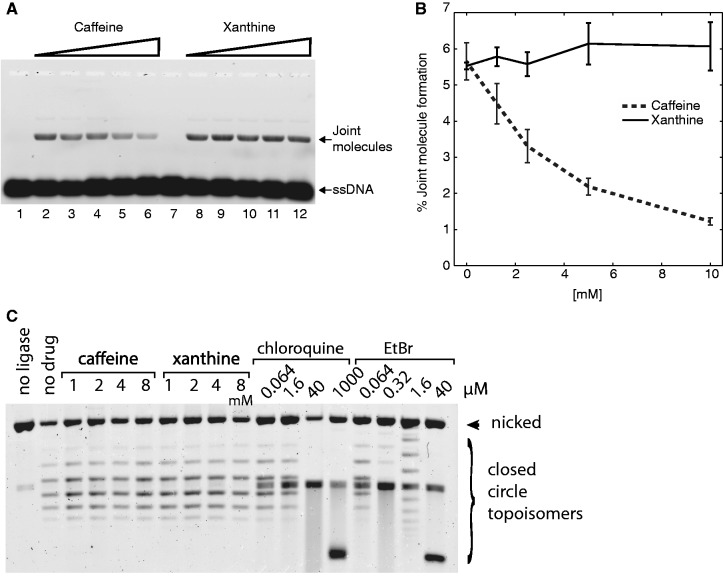
Inhibition of RAD51-catalyzed D-loop formation by caffeine. (**A**) Linear ssDNA, labeled fluorescently, was incubated with recombinant human RAD51 in the presence of different concentrations of caffeine or xanthine. Homologous supercoiled plasmid was then added to the formed RAD51-ssDNA filaments. ssDNA–dsDNA complexes (D-loops) were separated from the free ssDNA by agarose gel electrophoresis after deproteinization. A representative gel is shown. (**B**) Densitometric quantification of three independent gels as shown in panel (A). (**C**) Topoisomer-separating agarose gel analysis of the plasmid DNA that was nicked and re-ligated in the presence of the indicated concentrations of intercalating compounds.

As several independent approaches did not lend support for the hypothesis that the strong inhibition of GT by caffeine, which we demonstrated in three different assays, is mediated by the DDR, and other indirect pharmacological effects of caffeine could not be linked to GT inhibition either, we proceeded to test the possibility that caffeine affects the HR reaction directly. This possibility was suggested by previous studies in hamster cells that demonstrated that radiosensitization by caffeine is much less efficient in HR-deficient cells than in HR proficient (38) and that caffeine reduces the frequency of DSB-induced recombination between direct repeats (40). We further hypothesized that caffeine directly inhibits the core HR reaction either by disrupting protein–DNA interaction or by affecting ATPase activity and that a biochemical HR assay can recapitulate this effect. To test this hypothesis, we performed a D-loop assay—an *in vitro* reaction in which recombinant human RAD51 protein stimulates the formation of the pivotal HR intermediate between a linear ssDNA and a homology-containing supercoiled plasmid (51)—in the presence of caffeine or xanthine (Figure 4A and B). Caffeine strongly reduced the efficiency of D-loop formation, with a concentration dependence similar to that observed in the cellular GT assay, whereas xanthine, which did not inhibit GT *in vivo* (Figure 1), did not have such an effect.

The efficiency of D-loop formation in the assay we used is strongly dependent on the negative supercoiling of the plasmid template (51). It was suggested previously that caffeine can intercalate into and induce unwinding of DNA (52), suggesting a trivial explanation for our results. To rigorously determine whether caffeine and xanthine can unwind DNA, we incubated a circular singly nicked plasmid DNA with different concentrations of caffeine, xanthine or two known intercalating agents (ethidium bromide and chloroquine). The nick was subsequently ligated to fix any change in twist induced by intercalation. On removal of the intercalator, the induced changes in twist are converted in writhe, which can be analyzed by gel electrophoresis (Figure 4C). As expected, the distribution of the resulting topoisomers generated in the presence of low concentrations of chloroquine and ethidium bromide (nM to µM) were significantly different from the one obtained in the absence of the intercalator. This was not the case for caffeine and xanthine (Figure 4C), showing that at the concentrations used in our experiments (up to 8 mM), caffeine does not intercalate in double-stranded DNA (dsDNA). We therefore conclude that the reduction of D-loop formation efficiency in the presence of caffeine is not due to the topological effects on the template DNA.

Apart from the suggested intercalation, caffeine has been shown to bind DNA externally, changing the hydration shell of the DNA helix (53), which can explain the changes in affinity and specificity of the DNA-binding proteins in its presence (54). To test whether caffeine suppresses RAD51-catalyzed D-loop formation by disrupting RAD51-DNA filament formation, we used EMSA and found that RAD51 bound DNA with the same efficiency in the presence of up to 10 mM caffeine as without the drug (Supplementary Figure S2). The DNA-stimulated ATPase activity of RAD51 was also largely unaffected by caffeine (data not shown).

### Caffeine promotes non-productive nucleoprotein filament interactions

**Figure 5.**
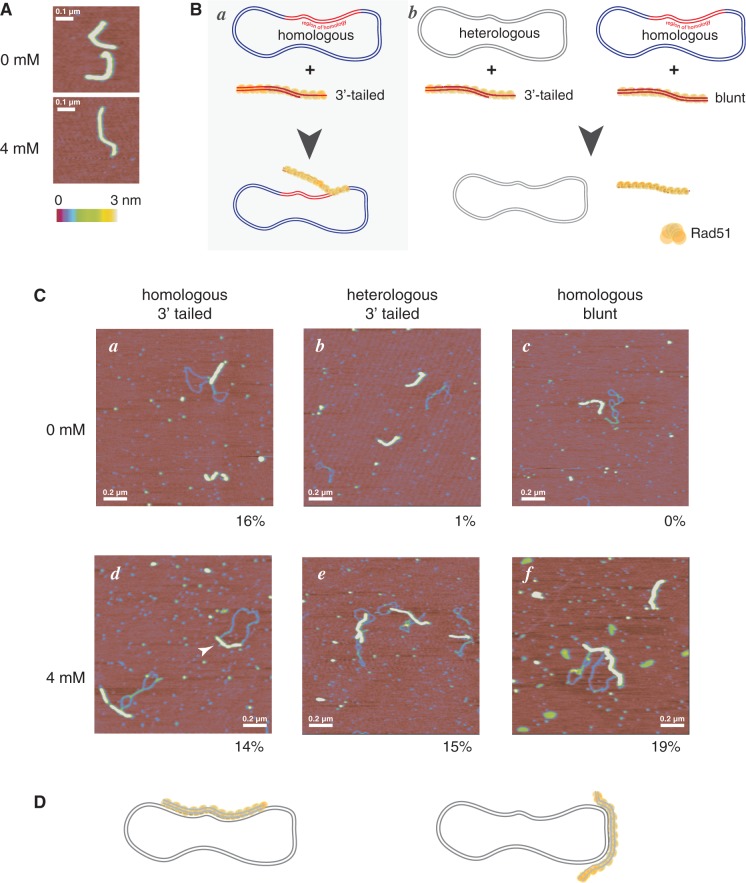
Architecture of the RAD51-catalyzed joint molecule formation in the presence of caffeine. (**A**). SFM images of RAD51 filaments formed on dsDNA in the presence or in the absence of 4 mM caffeine. (**B**) Schematic diagram of DNA substrates and their arrangement in product of homologous paring in SFM studies. The structure of the joint molecule formed in the reactions containing 3′-tailed RAD51 filament and homologous circular DNA is depicted schematically; intermediates in strand exchange include only the ssDNA region of the filament involved in pairing (*a*). In reactions containing blunt linear DNA lacking ssDNA in the filament or heterologous circular DNA joint molecules do not form (*b*). (**C**) SFM images of the joint molecules formed between the RAD51 filaments on 3′-tailed (*a,b,d,e*) or blunt (*c,f*) linear DNA and homologous (*a,c,d,f*) or heterologous (*b,e*) circular DNA in the absence (*a,b,c*) or in the presence of 4 mM caffeine (*d,e,f*). Numbers under the images indicate the fraction of the RAD51 filaments involved in joint molecule formation. (**D**) Schematic representation of the structure of the aberrant joint molecules formed in all filament-substrate combinations in the presence of 4 mM caffeine. Extensive pairing independent of ssDNA and homology was observed. In all SFM images, color indicates height from 0 to 3 nm as shown in the color scale under (A).

In an attempt to reveal a particular step in the mechanism of HR at which caffeine exhibits its inhibitory effect, we studied the RAD51-catalyzed strand invasion reaction at a single molecule level using scanning force microscopy (SFM). We first compared the structure of the RAD51 filaments assembled on 3′-single-strand tailed linear duplex DNA in the absence or in the presence of 4 mM caffeine. In agreement with the EMSA results, RAD51 binding to DNA as well as the appearance of the nucleoprotein filaments were unaffected by caffeine (Figure 5A). There was no difference in the length and height of the filaments formed in the absence or in the presence of caffeine (data not shown). Next, we analyzed the effect of caffeine on the architecture of joint molecules by SFM (34). Joint molecules were formed between filaments on either 3′-tailed or blunt-ended linear DNA and circular substrates with or without homology (Figure 5B). Without caffeine, joint molecules, as previously characterized extensively (34), were detected only in the reactions containing RAD51 filaments formed on 3′-tailed linear substrate and homologous circular DNA (16% of 261 filaments observed were paired in joint molecules at the position of homology, see examples in Figure 5C, *a*). Substitution of heterologous for homologous circular DNA or blunt-ended linear for 3′-tailed DNA did not result in joint molecule formation (only 1% of 221 heterologous filaments and 0% of 163 double-stranded filaments were associated non-specifically with circular DNA, Figure 5C, *b* and *c*). The length of the paired region in most of the joint molecules suggested that only the 3′ single-stranded tail was involved in homologous pairing.

In sharp contrast, when 4 mM caffeine was added to the reaction, interactions between the nucleoprotein filaments and the circular templates were observed for all three conditions (15% of 122 heterologous filaments and 19% of 42 double-stranded filaments were observed interacting non-specifically with the circular DNA, Figure 5C, *e* and *f*). These interactions often involved most of the filament length (as depicted in Figure 5D), suggesting that for the 3′-tailed linear substrate pairing clearly extended beyond the region of ssDNA. Of the joint molecules formed in the presence of caffeine, 45% of the 55 observed examples had filaments paired to dsDNA over a length much longer than the single-stranded region of the filament. This is in striking contrast to the reactions done in the absence of caffeine, where 0% of 43 joint molecules had paired regions longer than expected, and all were within the length corresponding to the ssDNA, as also observed previously (34). For comparison, see Figure 5C, *a* and *d* with showing a filament paired with dsDNA only at one end corresponding to length of the ssDNA region and *d* displaying an example indicated with white arrow filament paired with dsDNA for entire length including dsDNA in the filament.

Thus, in the presence of caffeine, RAD51 nucleoprotein filaments formed stable interactions with dsDNA independent of homology (all examples in Figure 5C, *e*) and single-stranded tails (all examples in Figure 5C, *f*). We conclude that the decrease in the efficiency of D-loop formation and thereby HR in the presence of caffeine is due to caffeine-induced unproductive interactions of the RAD51 nucleoprotein filament with non-homologous DNA.

## DISCUSSION

### The DDR and GT

We investigated whether introduction of numerous DSBs in the form of linearized targeting construct DNA molecules during a GT procedure triggers the DDR and whether the DDR affects the efficiency of GT and thus may be an attractive target for the improvement GT efficiency. Electroporation of ES cells with linear targeting DNA constructs did not cause alterations in the cell cycle progression that would be expected for a potentially strong DDR (Figure 2). Among several approaches that modulated the DDR, treatment with caffeine and several other alkylxanthines, and inhibition of ATR and its downstream target kinase Chk1 had a strong negative effect on GT by HR (Figures 1, 2C and 3). Further investigation of GT suppression by caffeine became the main focus of the study and revealed direct inhibition of HR as the most likely mechanism (Figures 4 and 5, discussed later in the text).

Based on the experiments with specific inhibitors and genetically deficient cells, we could rule out the requirement in GT of ATM and DNA-PKcs, the two kinases that are primarily responsible for DSB signaling and expected to be activated by the transfected linear DNA. Considering this, strong GT suppression by the inhibitors of ATR and its downstream target Chk1 was rather surprising. ATR is mainly activated by single-strand gaps that can arise during replication or nucleotide excision repair; therefore, it is interesting to consider why ATR rather than ATM and DNA-PKcs would be activated by the transfected DNA, and why this activation would be essential for GT.

We suggest that the DDR could be involved in GT even if it is not triggered by the transfected DNA. Instead, its activation could originate from GT-associated events in the genomic DNA of the targeted locus. Although ends of the targeting construct are recombinogenic (linear DNA targets more efficiently than circular), the targeted locus must also play an active role in the process. This follows from the two well-established observations. First, induction of a DSB can increase GT frequency at the locus by orders of magnitude, much more so than the linearization of the targeting construct. Second, different regions of the genome are targeted with different efficiency (1,55–57). For example, in mouse ES cells, the average targeting efficiency is 1–5% but varies in a broad range: some loci, such as Rad54 used in our study, are targeted with a frequency of 30–60%, whereas at other loci, recombinants cannot be obtained. Similar locus effects are observed in other species where GT was attempted, including protozoa, fungi and plants (58). One possible explanation can be differences chromatin accessibility. This predicts that transcriptionally inactive heterochromatic regions should be targeted at lower efficiency, but no such correlation could be established. In addition, we have found that large variations in GT efficiency can exist within the same transcription unit (AZ, unpublished). An alternative explanation, which we favor, is that GT must be triggered by some local repair event—a nick, a replication stall, or a DSB resulting from those—which occur with different frequencies across the genome. These local effects are highly likely to involve the DDR.

We suggest that the strong negative effect of the ATR and Chk1 inhibitors on GT is due to the suppression of the recombination-initiating event at the targeted locus. Our observation that ATR inhibitor suppresses GT when circular DNA is used supports this interpretation (Figure 2C). Linear DNA is much less stable in the cell than circular, and possible substrates for ATR activation should arise from it at a higher rate, for example by partial degradation at the ends. It is therefore expected that if the ATR-dependent process required for GT is initiated by the transfected DNA, its inhibition will be much lower in the experiments involving circular DNA. We however observed substantial inhibition of GT in cells electroporated with circular Rad54-GFP targeting construct and treated for 4 h with the ATR inhibitor, i.e. following the same protocol as used in the experiments with the linearized construct. Considering the much wider time window available for recombination with the circular DNA owing to its longer persistence, this inhibition is not compatible with ATR activation by the construct.

We conclude that, in contrast to previous studies with microinjection, the ends of the linear DNA delivered by mass-transfection methods do not trigger the DDR with the same efficiency as DSBs in genomic DNA, and that the DDR in reaction to transfected DNA plays a minor role, if any, in GT by commonly used protocols. Possible explanations for this may be the changes that the DNA undergoes on the way to the nucleus from the cytoplasm, where it is delivered by most mass-transfection methods, including electroporation (59), or the differences in chromatinization of the substrate.

### Caffeine directly inhibits HR

Our most intriguing finding is the identification of caffeine and related alkylxanthines, but not xanthine or hypoxanthine, as inhibitors of GT by HR. The inhibition can be efficient (>80%), reversible (pre-treatment before electroporation has no effect) and rapid (extended incubation has nearly the same effect as a 4-h treatment) (Figure 1). These features suggest that caffeine affects the HR reactions directly, rather than by inducing changes in the cell physiology. Importantly, using an *in vitro* HR assay with a central protein of human HR, we could re-capitulate not only the inhibitory effect of caffeine but also the difference between caffeine and xanthine, with a concentration dependence similar to that observed in the cellular GT assay (Figures 4 and 5). As intracellular concentration of caffeine equilibrates with the extracellular concentration within minutes after addition (60), this, combined with the negative results from our extensive attempts to link GT inhibition to other known physiological effects of caffeine, strongly suggests that RAD51 can be the target for caffeine during GT *in vivo*.

### Linking caffeine to HR

Inhibitory effect of caffeine on recombination and recombinational repair in bacteria (61–63), yeast [meiotic (64) and mitotic (65)] and *Drosophila* (66) have been documented. As for mammalian cells, several previous studies suggested an effect of caffeine on homology-dependent repair (HDR) based on the use of the HDR assays and HR mutants in the context of induced damage (ionizing radiation or endonuclease) (38,40,67). Caffeine has been reported to suppress HR-mediated repair of a nuclease-induced DSB (40). However, in the system used, the reduction in HR products (GFP-expressing cells) could also be explained by factors other than inhibition of core HR machinery; for example, caffeine could reduce the efficiency of break induction or reduce the survival of the subpopulation of cells where the break was induced at sufficient levels. Furthermore, another study using a similar I-SceI-inducible HDR substrate found no effect of caffeine and other PIKK and CDK inhibitors on HR (68).

Although our data demonstrate efficient inhibition of HR-mediated GT, we find it unlikely that caffeine inhibits all forms of HR with equal efficiency. First, it had a relatively small effect on the plating efficiency of ES cells, while core HR is essential for replication (69,70). The mechanism revealed by our biochemical and SFM experiments (discussed later in the text) suggests that homology search by the RAD51 nucleoprotein filament becomes inefficient in the presence of caffeine. We surmise that HR pathways that are less dependent on genome-wide homology search, such as restoration of replication fork or recombination with the sister chromatid in the state of cohesion, will be more immune to the inhibitory action of caffeine than GT. Second, even in prolonged treatments with high concentrations of caffeine (8 mM) and other alkylxanthines, ∼20% of the GT events are unaffected, suggesting that this fraction may use a caffeine-resistant pathway.

### The RAD51 nucleoprotein filament as a target of caffeine

Our biochemical and microscopy experiments reveal a specific step in the HR reaction targeted by caffeine. RAD51, and other DNA-binding proteins implicated in HR (RPA and MRN), can bind DNA with the same apparent affinity in the presence of up to 8–10 mM caffeine (Figure 4 and Supplementary Figures S2 and S3). We also did not see any effect of caffeine on mismatch-dependent nicking by recombinant *E. coli* MutS/L/H complex, or plasmid DNA digestion by several restriction endonucleases, including I-SceI, which has an 18-bp long recognition sequence (data not shown). At the same time, RAD51-catalyzed strand invasion was inhibited due to non-productive interactions of the RAD51 filament with double-stranded partner DNA (Figure 5).

We propose that the difference in the strengths and perhaps complexity of protein–DNA interactions can explain this distinction. During homology search, a recombinase, such as RAD51 or *E. coli* RecA, has to sample a genome-wide sequence space, probing the vast heterologous background. Encounters with heterologous sequence therefore must rely on transient and weak protein–DNA interactions (71,72). In this respect, the effect of caffeine on RAD51 is similar to the early observations by Selby and Sancar (54) on *E. coli* DNA photolyase and (A)BC excinuclease. In case of (A)BC excinuclease, 10 mM caffeine did not inhibit the ATPase activity of UvrA, which needs to scan the DNA to identify the lesion, and promoted its binding to DNA while reducing the specificity.

In addition to the site occupied by the filament-containing DNA molecule, there are two other DNA-binding sites on RAD51 (73)—the secondary DNA-binding site, common to both RAD51 and RecA, and the N-terminal domain, not present in RecA. Mutation of critical residues in the N-terminal domain leads to primary DNA-binding defects, which can be revealed by EMSA assays with ss- and dsDNA, and in the case of yeast Rad51, the ATPase activity (74,75). As we detect no effect of caffeine on human RAD51 in similar assays, it is unlikely that the N-terminal domain is its target. The secondary DNA-binding site, on the other hand, comes into play during the interaction of the filament with the incoming dsDNA, the step that is affected by caffeine in our SFM experiments. A recent study using dual-molecule techniques provided mechanistic insights into the function of the secondary DNA-binding site in the prototypical recombinase RecA (71). The secondary DNA-binding site interaction with the incoming dsDNA is much weaker than with ssDNA, regardless of whether the filament was formed on ss- or dsDNA, and it can be strongly stimulated by the unwinding of the incoming dsDNA. Caffeine (and other methylxanthines) have long been known to bind ssDNA (76) or partially denatured dsDNA (77), lower the dsDNA melting temperature (78), stimulate the digestion of dsDNA by S1 nuclease that has strong preference for denatured (ss) DNA (79) and, in general, posses solubilizing properties (80). From these findings, we hypothesize that caffeine may stabilize the interaction of the incoming dsDNA with the filament by facilitating its melting and/or by preventing re-annealing of the ssDNA that has entered the secondary DNA-binding site. Alternatively, binding of caffeine to DNA via an external mode described by Fritzsche *et al.* (53) may increase its affinity to the RAD51 secondary DNA-binding site without melting the helix, but in the absence of detailed structural information, it is currently unclear how this stimulation would be achieved.

### Caffeine, PIKKs (ATM/ATR/DNA-PKcs) and weak protein–DNA interactions

Early models explaining caffeine radiosensitization by its direct effect on protein–DNA interactions were eclipsed by the demonstration of the *in vitro* inhibition of the ATM, ATR and DNA-PKcs kinases, and currently the checkpoint-mediated mechanisms are nearly universally used in the literature to explain the effects of caffeine. We could not re-capitulate the inhibitory effect of caffeine on GT using alternative approaches to perturb the DDR, and we did not observe the inhibition of ATM and ATR targets’ phosphorylation in the presence of caffeine *in vivo* (Figure 2). Consistent with our finding, a number of studies reports phosphorylation of ATM/ATR/DNA-PKcs substrates in the presence of millimolar concentrations of caffeine; most notably: Chk1 (39,81,82), RPA2 (83,84), Chk2 (82,85), as well as increased γH2AX in HeLa (86) and mouse ES cells (41).

Although the ability of caffeine to inhibit ATM/ATR/DNAPKcs-dependent phosphorylation cascades in most circumstances is well documented, the existence of such exceptions puts in question the proposed mechanism—direct competitive inhibition of the kinase active site—suggested based on chemical similarity between caffeine and ATP. Indeed, the competitive inhibition mechanism is not supported by the only study in which it was tested (87). Interestingly, the presence of (damaged) DNA is required not only for activation but also for sustained high activity of ATM (88), ATR (89) and DNA-PK (90). This raises an intriguing possibility that this DNA-dependent stimulation, rather then the kinase activity itself, is the target of caffeine inhibition.

Our study demonstrates that direct affects on HR have to be taken into account when interpreting the physiological effects of caffeine and other alkylxanthines. According to the model we propose, HR inhibition by caffeine is due non-productive homology search by the Rad51 filament. Rapid, efficient and reversible inhibition of HR-mediated GT by caffeine provides a powerful tool to study this elusive process.

## SUPPLEMENATARY DATA

Supplementary Data are available at NAR Online: Supplementary Figures 1–3 and Supplementary References [91,92].

## FUNDING

TOP grant from the Netherlands Organization for Scientific Research (NWO) Chemical Sciences [grant number VICI 700.56.441]; Marie Curie Intra-European Fellowship [grant number FP7-221069]; Reintegration Grant [grant number FP7-276898 to H.S.]; and the Netherlands Genomics Initiative/NWO, as well as an ECHO grant from NWO-Chemical Sciences. The research leading to these results has received funding from the European Community’s Seventh Framework Programme (FP7/2007-2013) under [HEALTH-F2-2010-259893]. Funding for open access charge: The research leading to these results has received funding from the European Community's Seventh Framework Programme (FP7/2007-2013) under [grant agreement No. HEALTH-F2-2010-259893].

*Conflict of interest statement.* None declared.

## Supplementary Material

Supplementary Data
