# Supplementary material for: Caffeine suppresses homologous recombination through interference with RAD51-mediated joint molecule formation
Source: Nucleic Acids Res. 2013 May 10;41(13):6475–89. doi: 10.1093/nar/gkt375 (PMC3711438; doi:10.1093/nar/gkt375)
Supplement: Supplementary Data [file supp_41_13_6475__index.html]

Caffeine suppresses homologous recombination through interference with RAD51-mediated joint molecule formation — Caffeine suppresses homologous recombination through interference with RAD51-mediated joint molecule formation — Supplementary Data 

# Caffeine suppresses homologous recombination through interference with RAD51-mediated joint molecule formation

## Supplementary Data

files

**Files in this Data Supplement:**

- Supplementary Data - pdf file
