# Supplementary material for: Caffeine suppresses homologous recombination through interference with RAD51-mediated joint molecule formation
Source: Nucleic Acids Res. 2013 May 10;41(13):6475–89. doi: 10.1093/nar/gkt375 (PMC3711438; doi:10.1093/nar/gkt375)

Supplementary Figure Legends

Supplementary Figure 1. Rad54-GFP and Rosa26-ßgeo gene targeting assays

(**A**) Rad54-GFP knock-in GT assay. Stable integration, either by HR or randomly, of the targeting knock-in construct confers puromycin resistance. Correct HR with the Rad54 locus leads to the expression of a Rad54-GFP fusion protein (91, 92). The Rad54-GFP targeting construct carries a puromycin resistance gene under a PGK promoter, and relative efficiency of GT is determined by FACS as a fraction of GFP-positive cells among puromycin-resistant transformants. Although the reporter can also be expressed if the targeting construct integrates by illegitimate recombination in a suitable transcription and splicing context, this scenario is relatively unlikely, and using Southern blotting we determined that >95% of the reporter-expressing cells are due to GT by HR. A similar observation was made for the Rosa26-βgeo GT assay described below. (**B**) The Rosa26-βgeo knock-in assay is based on the same principles. The number of G418-resistant colonies reflects the absolute efficiency of GT. The frequency of random integration in this assay can be measured by co-transfection of 2 μg linear DNA containing puromycin-resistance gene under a PGK promoter with 10 μg of the Rosa26-βgeo targeting construct. This knock-in approach allows monitoring targeting events in genes whose native products (or absence thereof) are not directly selectable without the need for tedious screening by Southern blotting or PCR.

Supplementary Figure 2. Effect of caffeine on DNA binding by RAD51

Reaction mixtures for electrophoretic mobility shift assays containing RAD51 and a either a double-stranded (panel A) or single-stranded (panel B) fluorescently labeled oligonucleotide (AF488SK3ds or AF488SK3ss) and human RAD51 (300 nM) were assembled in reaction buffer in the presence of caffeine (final concentrations: 0, 0.5, 1, 5 and 10 mM, respectively). Samples

were incubated for 10 min at 37°C, in a final volume of 20 µl. DNA and protein-DNA complexes were separated on a 5% non-denaturing polyacrylamide gel running in 0.5× TB buffer at 4°C.

Supplementary Figure 3. EMSAs with RPA and MRN.

EMSA of ssDNA-binding of RPA (panel A) and MRN (panel B) protein complexes in the presence or absence of caffeine. RPA (2.5 nM) and MRN (100 nM) protein complex were incubated with 1 nM 70 nt ssDNA oligonucleotide labeled with Alexa Fluor 532 at the 5' end. Protein-ssDNA complexes and unbound ssDNA were separated by electrophoresis through a native polyacrylamide gel and visualized using a Typhoon scanner. Caffeine showed no effect on formation of RPA or MRN protein complexes with the ssDNA at any of the caffeine concentrations tested.

Supplementary Figure 1

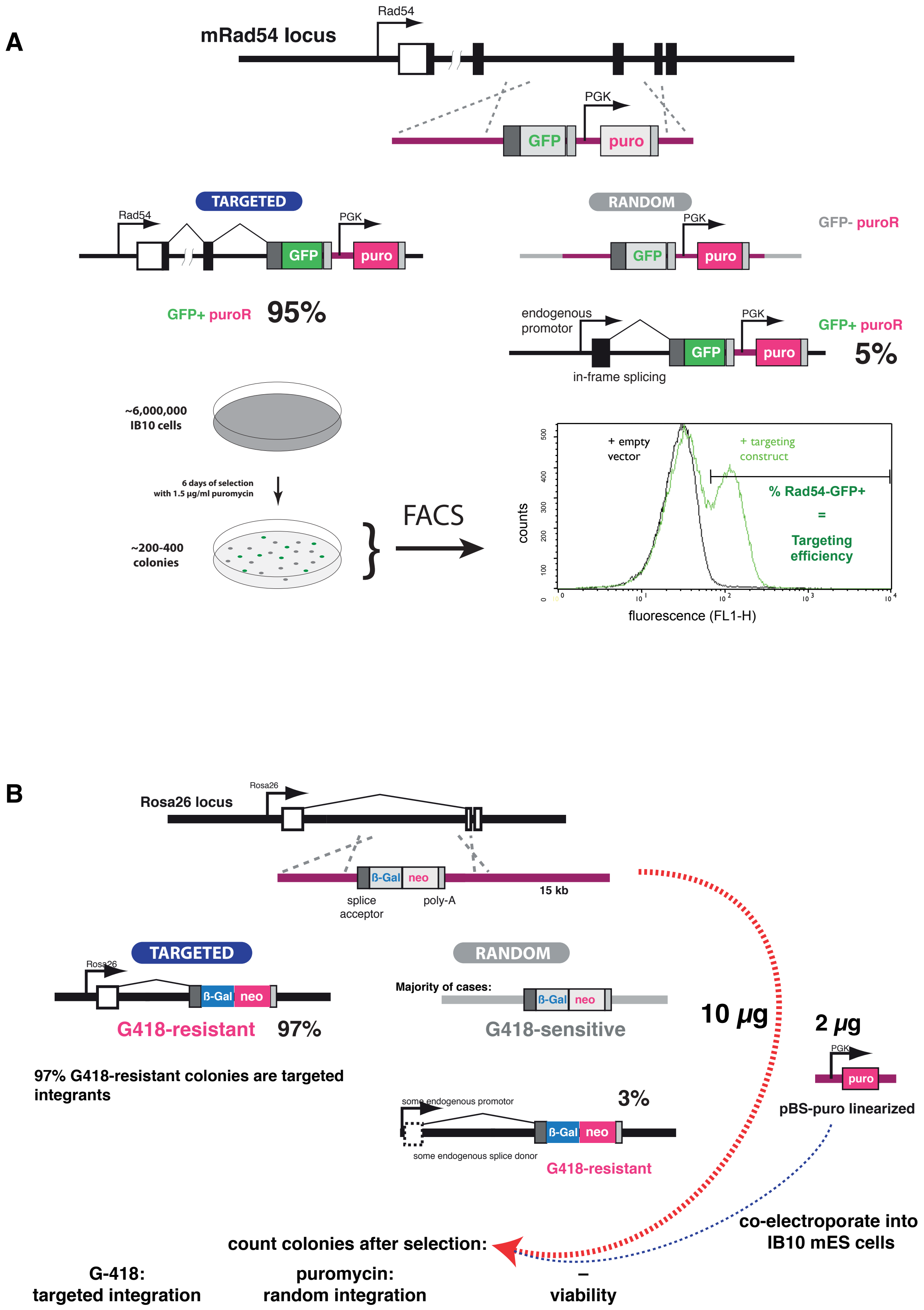

Supplementary Figure 2

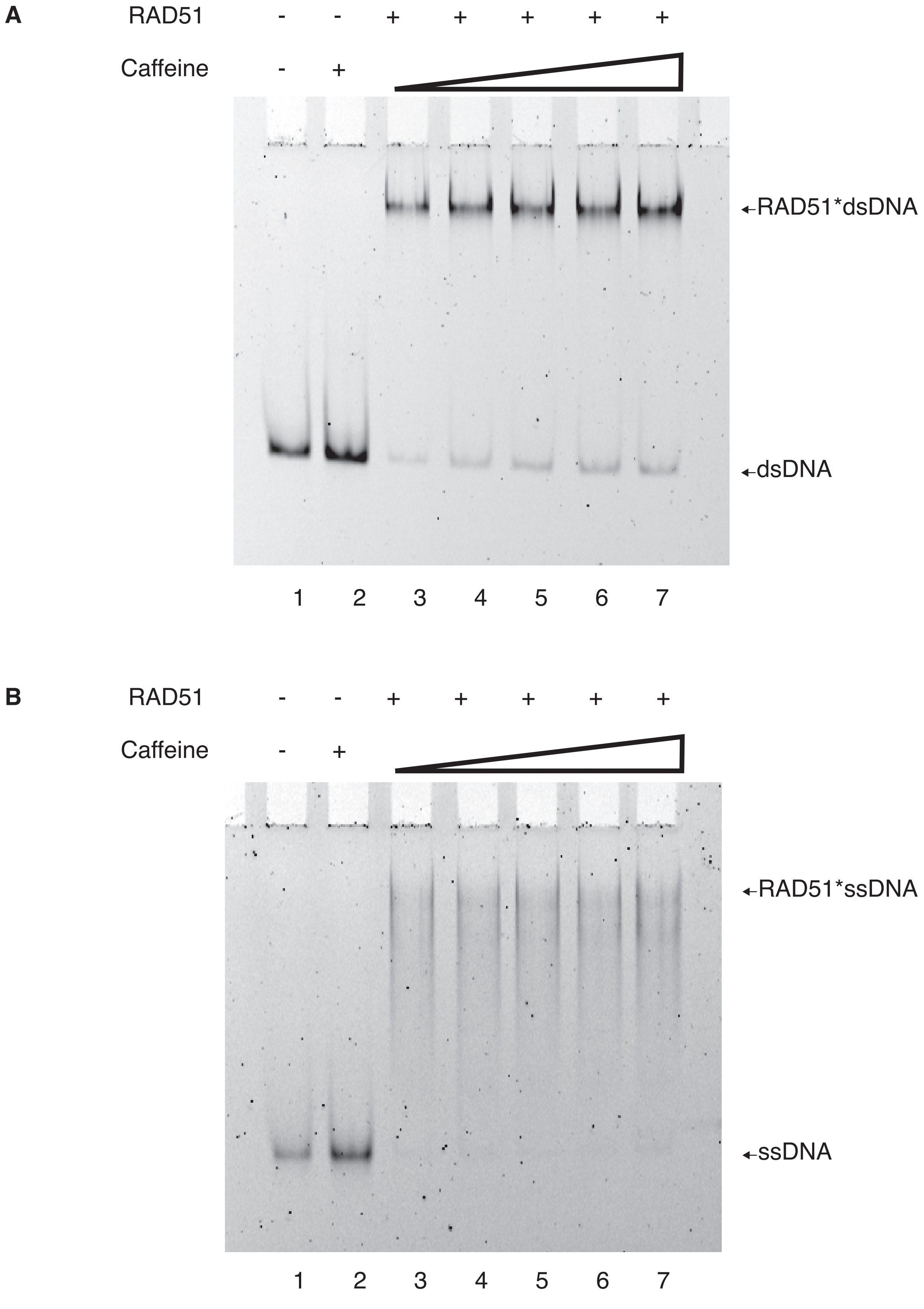

Supplementary Figure 3

A

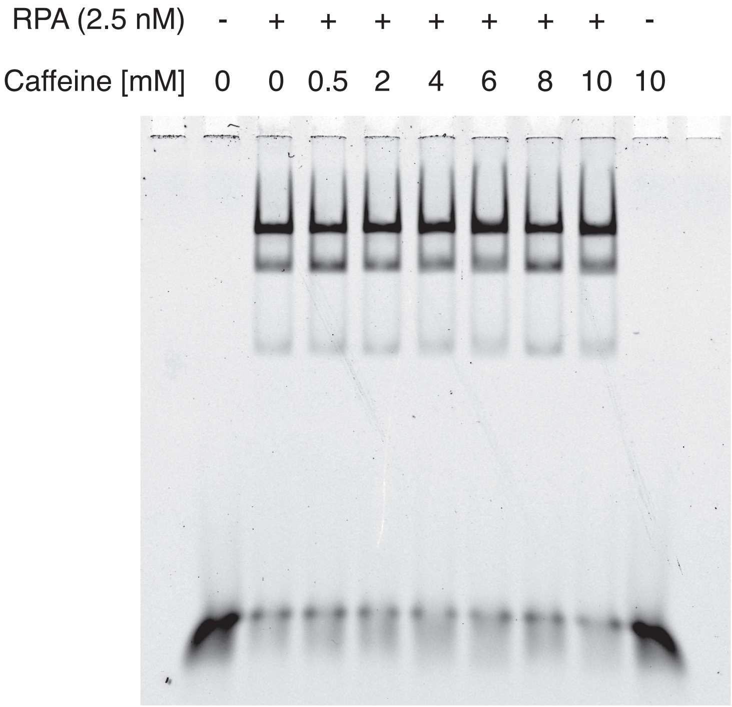

B

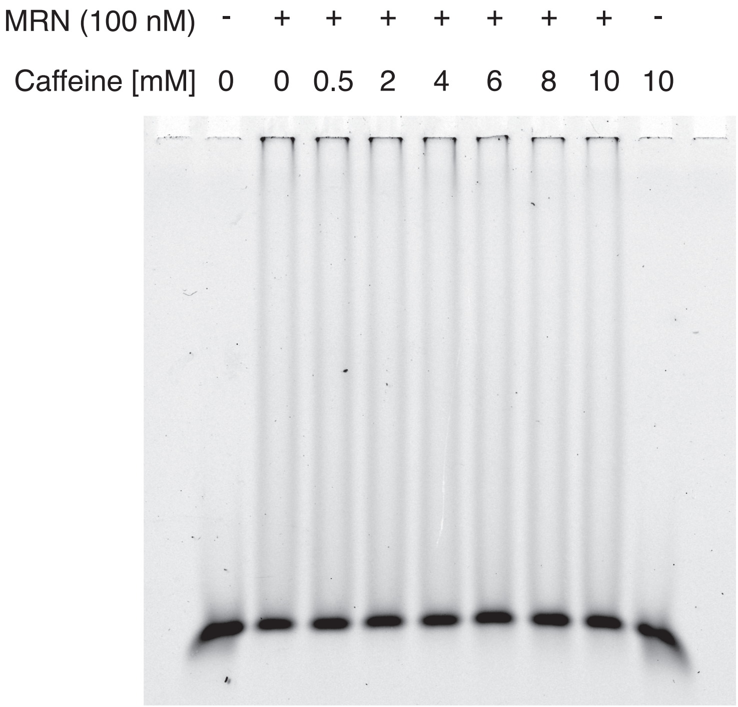

Supplement: Supplementary Data [file supp_gkt375_nar-00186-d-2013-File007.pdf]
